# Macrophage-derived SHP-2 inhibits the metastasis of colorectal cancer via Tie2-PI3K signals

**DOI:** 10.32604/or.2023.028657

**Published:** 2023-04-10

**Authors:** XUELIANG WU, SHAOYU GUAN, YONGGANG LU, JUN XUE, XIANGYANG YU, QI ZHANG, XIMO WANG, TIAN LI

**Affiliations:** 1 Department of Gastrointestinal Surgery, Tianjin Medical University Nankai Hospital, Tianjin, 300100, China; 2 93868 Troop of the Chinese People’s Liberation Army, Yinchuan, 750021, China; 3 Clinical Laboratory, Hebei General Hospital, Shijiazhuang, 050051, China; 4 Department of General Surgery, The First Affiliated Hospital of Hebei North University, Zhangjiakou, 075000, China; 5 Tianjin Key Laboratory of Acute Abdomen Disease Associated Organ Injury and ITCWM Repair, Institute of Integrative Medicine for Acute Abdominal Diseases, Integrated Chinese and Western Medicine Hospital, Tianjin University, Tianjin, 300100, China; 6 School of Basic Medicine, Fourth Military Medical University, Xi’an, 710032, China

**Keywords:** SHP-2, Tie2, PI3K, Akt/mTOR signaling, Colorectal cancer, Liver metastasis, Macrophages

## Abstract

This research aimed to explore the influence of Src homology-2 containing protein tyrosine phosphatase (SHP-2) on the functions of tyrosine kinase receptors with immunoglobulin and EGF homology domains 2 (Tie2)-expressing monocyte/macrophages (TEMs) and the influence of the angiopoietin(Ang)/Tie2-phosphatidylinositol-3-kinase (PI3K)/protein kinase B (Akt)/mammalian target of rapamycin (mTOR) (Ang/Tie2-PI3K/Akt/mTOR) signaling pathway on the tumor microvascular remodeling in an immunosuppressive microenvironment. *In vivo*, SHP-2-deficient mice were used to construct colorectal cancer (CRC) liver metastasis models. SHP-2-deficient mice had significantly more metastatic cancer and inhibited nodules on the liver surface than wild-type mice, and the high-level expression of p-Tie2 was found in the liver tissue of the macrophages’ specific SHP-2-deficient mice (SHP-2MAC-KO) + planted tumor mice. Compared with the SHP-2 wild type mice (SHP-2WT) + planted tumor group, the SHP-2MAC-KO + planted tumor group experienced increased expression of p-Tie2, p-PI3K, p-Akt, p-mTOR, vascular endothelial growth factor (VEGF), cyclooxygenase-2 (COX-2), matrix metalloproteinase 2 (MMP2), and MMP9 in the liver tissue. TEMs selected by *in vitro* experiments were co-cultured with remodeling endothelial cells and tumor cells as carriers. It was found that when Angpt1/2 was used for stimulation, the SHP-2MAC-KO + Angpt1/2 group displayed evident increases in the expression of the Ang/Tie2-PI3K/Akt/mTOR pathway. The number of cells passing through the lower chamber and the basement membrane and the number of blood vessels formed by cells compared with the SHP-2WT + Angpt1/2 group, while these indexes were subjected to no changes under the simultaneous stimulation of Angpt1/2 + Neamine. To sum up, the conditional knockout of SHP-2 can activate the Ang/Tie2-PI3K/Akt/mTOR pathway in TEMs, thereby strengthening tumor micro angiogenesis in the microenvironment and facilitating CRC liver metastasis.

## Introduction

Colorectal cancer (CRC) originates from the epithelial cells of the colonic or rectal mucosa. The incidence of rectal cancer occupies 60%–70% of the total of colorectal cancer, and rectal adenocarcinoma (READ) accounts for over 85% of rectal cancer, which is commonly seen among elderly people and people with poor lifestyles or eating habits [1].

CRC metastasis can be classified into lymph node metastasis, hematogenous metastasis, and direct invasion. Specifically, liver metastasis and lung metastasis are common hematogenous metastasis [2–4]. The former is the main killer of CRC patients, as well as a serious challenge in the management of CRC [5,6]. Tumor metastasis is an extremely complicated process accompanied by a series of changes in genetics and the tumor microenvironment (TME). Recently, targeting TME has become a new approach to preventing tumor recurrence and metastasis. TME is a cell population composed of tumor cells and many kinds of stromal cells they recruit, such as T cells, macrophages, and composed of tumor cells and many kinds of stromal composed of tumor cells and many kinds of stromal cells they recruit, such as T cells, macrophages, and fibroblasts [7–9]. It has been found recently that tumor-associated macrophages (TAMs) are a major part of both the tumor immunosuppressive microenvironment and the stromal cells infiltrating the TME [10]. In mice breast cancer models, there is a type of tumor-infiltrating myeloid cells expressing the angiopoietin (Ang) receptor and tyrosine kinase receptors with Tie2 under tumor-bearing environments, namely Tie2-expressing monocyte/macrophages (TEMs) [11]. It has been identified in previous studies that Ang1/Ang2-Tie2 pathway regulates TEMs and is involved in tumor immunosuppression and micro angiogenesis [12–16]. In addition, the phosphatidylinositol-3-kinase (PI3K)/protein kinase B (Akt)/mammalian target of Rapamycin (mTOR) (PI3K/Akt/mTOR) pathway mediated by Ang1/Ang2-Tie2 is the main channel involved in TEMs-related tumor micro angiogenesis and immunosuppressive microenvironment. As one of the most pivotal pathways in regulating the cell cycle, the PI3K/Akt/mTOR pathway not only participates in several biological processes including cell proliferation, differentiation, migration, apoptosis, and adhesion has also been extensively researched and applied to the development of novel antitumor agents [16,17].

The abnormal or hyper-activation of tyrosine phosphatase and tyrosine kinase plays a critical role in the progression of various diseases, especially in a tumors. SHP-1 and SHP-2 are major components of tyrosine phosphatases and unlike SHP-1, which directly dephosphorylation of tyrosine kinases, the SHP-2’s roles exhibit the diversity and uncertainty [18–23], thereby the effects of SHP-2 need to be investigated to clarify its mechanisms. As revealed by previous research, Src homology-2 containing protein tyrosine phosphatase (SHP-2), an intracellularly expressed non-receptor tyrosine phosphatase with protein tyrosine phosphatase activity, plays a crucial role in the regulation of pattern recognition receptors (PRRs) [24], however, SHP2 in macrophages showed different or even opposite roles in various diseases, for example, the myeloid SHP2-knockout, amplified the inflammatory responses under lipopolysaccharide stimulation [25] and promoted STAT6 mediated M2-polarization and suppression of inflammation in pulmonary fibrosis [26]. Moreover, SHP2 under stimulation in various tumors or different diseases may activate or inactivated, indicating the complexity and diversity of SHP2 [27–33]. Thereby, the research about SHP2 needs to be tested and verified by consequential cellular or animal experiments in various cell types or diseases. In addition, it also acts as a negative regulator of Tie2 phosphorylation, and its low expression is correlated with poor tumor differentiation and tumor-node-metastasis (TNM) stage [34,35]. The Tie2+ macrophages play a pivotal role in tumor’ neovascularization by activating the PI3K/Akt/mTOR signaling pathway and result in increased expression of angiogenic factor (VEGF, COX-2, MMP-2/-9) in TEMs. As the ligands for Tie2, Ang-1/2 is produced by angiogenic tumor vessels and fully expressed in the tumor microenvironment, therefore, forming a vicious cycle to promote tumor progression. However, the exact mechanisms of SHP-2 in TEMs are ambiguous and our studies were carried on to explore and clarify these mechanisms.

## Test Methods

### Construction of the animal model

Through the Cre-LoxP gene targeting system, cell-specific SHP-2-deficient mice were screened from the hybrid progeny of Lyz2-Cre+/− mice and SHP-2 folx/+C57BL/6 mice: Strain #:025758, and transgenic to Balb/c background for 9 generations for research use. In macrophage-specific SHP-2-deficient mice and wild-type mice, CRC cells CT26 were used to construct animal models of CRC liver metastases [3]. The cancer cell suspension with a measured cell concentration of 2.5 × 107/mL of CT26 cells was slowly injected into the spleen of mice. About 3–5 min later when the injection site became white and swollen, the injector was withdrawn and the needle hole was immediately pressed with an alcohol cotton ball until no active bleeding was found. After the muscle and skin were sutured intermittently and the abdomen was closed, the mice were transferred to an incubator to keep warm and subjected to intraperitoneal injection with 0.1 mL of 1% piperacillin. Then they were fed with glucose saline after resuscitation. The SHP-2WT and SHP-2MAC-KO mice injected with physiological saline were used as control groups, and the SHP-2WT and SHP-2MAC-KO mice injected with CT26 cells were used as tumor-planted groups. Three weeks later, the mice were killed by carbon dioxide, immediately after which the laparotomy was performed to observe the number, size, and location of metastatic cancer nodules on the liver surface. In addition, the metastases were fixed via formaldehyde, embedded in paraffin, and serially sectioned, followed by hematoxylin-eosin (HE) staining. Then the slices were observed under an optical microscope to clarify the situation of liver metastasis.

### HE staining

After deparaffinization and rehydration paraffin sections were stained with hematoxylin solution (Beijing Solarbio Technology Co., Ltd., Beijing, China) for 5 min followed by 5 dips in 1% acid ethanol (1% HCl in 70% ethanol) and then rinsed in distilled water. Then the sections were stained with eosin solution for 3 min and followed by dehydration with graded alcohol and clearing in xylene. The stained slices were scanned by motion digital slide assistant and analyzed by Image-Pro Plus 6.0 (Media Cybernetics, USA).

### Immunofluorescence

The slices were placed in 0.1 mol/L citrate antigen retrieval solution (pH = 6) for antigen retrieval, immersed in phosphate buffer saline (PBS), added with blocking serum dropwise, and then blocked in a wet box at room temperature for 1 h. After the blocking solution was wiped off by filter paper, the slices were washed with PBS twice and incubated with CD68 (Abcam, Cambridgeshire, UK, 1:80 dilution) and p-Tie2 primary antibodies (Abcam, 1:50 dilution) at an appropriate concentration in a wet box overnight at 4°C. After the recovery of the primary antibody and PBS washing, the slices were incubated again with fluorescent secondary antibodies away from light in a wet box at room temperature for 1 h. Then secondary antibodies were washed off thrice with PBS for 3 min each, followed by incubation with 4′,6-diamidino-2-phenylindole (DAPI) away from light for 15 min. After PBS washing, the slices were mounted with an antifade mounting medium and observed under a fluorescence microscope. Moreover, the Rabbit monoclonal of SHP-2 and mouse monoclonal of CD68 were used as primary antibodies and associated second antibodies as Goat Anti-Rabbit IgG H&L (Alexa Fluor® 555) and Rabbit Anti-Mouse IgG H&L (Alexa Fluor® 488) were used for the test of immunofluorescence double staining for human CRC specimens and all transwell plates were purchased from Corning Inc (NY, USA).

### Extraction of TEMs

Extraction of TEMs has used flow cytometry sorting technology in liver metastatic tissues of colorectal cancer. The hepatic tissues of tumor-bearing mice were isolated under sterile conditions, the blood stains were washed with D-PBS, and the tissues were cut into 1 mm 3 tissue blocks with sterile scissors. The tissue blocks were continuously stirred and digested in a mixer containing 0.2% type IV collagenase (No. C8160, Solarbio) and 0.25% trypsin (No. T1350, Solarbio) at 37°C, using a 50 ml conical bottle and a sterile small magnetic stirring rod for 5 min each time. The supernatant was collected after the digestion was terminated with the complete medium until the tissue mass disappeared. The supernatant collected after digestion was filtered with a 200-micron mesh screen, resuscitated with a cell staining buffer (1 × PBS containing 1% BSA), and refrigerated at 4°C. Cell staining and flow sorting: adjust the cell density to 1 × 106/100 μ l and add anti-F4/80-FITC (No. 11-4801-82, Invitrogen, Waltham, MA, USA,) and anti-Tie-2 to avoid light staining for 30 min. Cells were analyzed and sorted by BD FACSAria III sorted flow cytometry (Becton, Dickinson and Company, Franklin Lake, NJ, USA), and the photomultiplier tube (PMT) and adjustment compensation (FITC *vs*. APC) were optimized by monochromatic anti- F4/80-FITC anti-Tie-2 and negative control. Tumor-associated macrophages (TAMs) were purified by using the regional labeling of the F4/80+ and Tie-2+ cell populations.

### Primary TEMs’ cell culture and treatment

*In vitro*, the culture of TEMs by using flow cytometry sorting technology: macrophages were screened and extracted from cell-specific SHP-2-deficient and wild-type mice. The orbital blood of mice was centrifuged in a centrifuge tube at 2000 g for 20 min. After the upper plasma was removed, the lower cell pellet was obtained and resuspended using Hank’s solution into the single-cell suspension. Then, Angpt1/2 and Angpt1/2 + Neamine were added to TEMs sorted by flow cytometry from SHP-2WT and SHP-2MAC-KO mice’ liver metastatic tissues of colorectal cancer, respectively, to observe their influences on the functions of TEMs. All cells were classified by treatment methods into six groups: the SHP-2WT group, the SHP-2MAC-KO group, the SHP-2WT + Angpt1/2 group, the SHP-2MAC-KO + Angpt1/2 group, the SHP-2WT + Angpt1/2 + Neamine group,and the SHP-2MAC-KO + Angpt1/2 + Neamine group. Moreover, angiopoietin-1, angiopoietin-2 protein, mouse (HEK293, His) at 2 μg/mL (100 μl/well) can bind mouse TEK (Tie-2)-Fc. Neamine, a degradation product of neomycin, is a broad-spectrum aminoglycoside antibiotic, Neamine is an anti-angiogenesis agent targeting angiogenin. All reagents were purchased from MedChemExpress (MCE, NJ, USA).

### Transwell assay

Six groups of cells were co-cultured with MC38 and CT26 cells, respectively. Migration assay: 100 μL of cell suspension obtained by cell resuspension in the serum-free medium was added to the upper chamber of Transwell and 600 μL of the medium containing serum was added to the lower chamber for 48 h. Thereafter, the non-migrating cells on the top surface of the membrane were removed with cotton swabs, and the cells on the lower surface were fixed with paraformaldehyde. After rinsing, the fixed cells were stained with crystal violet, washed, and dried, followed by counting under an inverted microscope. Invasion assay: Matrigel diluted with the serum-free medium at a ratio of 1:8 was used to coat the upper surface of the bottom membrane of the Transwell chamber. Then the chamber was incubated in a 37°C incubator for 4 h so that Matrigel was polymerized into a gel for later use. The remaining steps were the same as those in the migration assay. All transwell plates were purchased from Corning Inc. (NY, USA).

### Wound healing assay

Six groups of cells were co-cultured with MC38 and CT26 cells, respectively. After digestion, the cells in the logarithmic growth phase were seeded into 24-well plates at a density of 1 × 10^5^ cells/mL and cultured in a serum-free medium for 6 h when the cell density reached 90%. Afterward, a 10 μL sterile pipette tip was used to make a scratch and the detached cells were gently removed by pre-warmed PBS. Then, the cells were treated with 50 ng/mL insulin for 48 h. Finally, the scratch width of cells was observed and measured at 0, 24, and 48 h, respectively, under an inverted phase-contrast microscope.

### Tube formation assay

Six groups of cells were co-cultured with C166 cells, and Matrigel was used to provide support. Then 100 μL of the cell suspension after digestion was put in a Matrigel-coated 96-well plate, marked, and routinely cultured in an incubator. They were observed and photographed under a microscope at 2–24 h of routine culture. In addition, five visual fields were randomly selected to observe and count the number of blood vessels formed by vascular endothelial cells.

### Digital western blotting

The protein was extracted and the protein concentration was determined by the BCA method. Configure DTT, according to the kit instructions, 5× MasterMix, Ladder, luminous liquid. We prepare the sample (final concentration is 0.5–3 μL), denature the prepared sample at 95°C for 5 min, cool it on ice for 5 min, mix it with vortex oscillation, and centrifuge. We configure the first antibody and add the prepared reagents into the plate according to the instructions, and centrifuge for 5 min. Take out the capillary tube, get on the machine, and start running tested by Jess multi-function automatic protein immunoblot quantitative analysis system (Protein simple company, California, USA). The primary antibodies are p-Tie2, t-Tie2, p-PI3K, t-PI3K, p-Akt, t-Akt, p-mTOR, t-mTOR, vascular endothelial growth factor (VEGF), cyclooxygenase-2 (COX-2), matrix metalloproteinase 2 (MMP2), MMP9 and glyceraldehyde-3-phosphate dehydrogenase (GAPDH). All first antibodies and second antibodies were purchased from Abcam and the data analysis with GAPDH as an internal reference.

### Extraction of primary tumor-associated macrophages for Western blot in vivo

**Figure S1:**
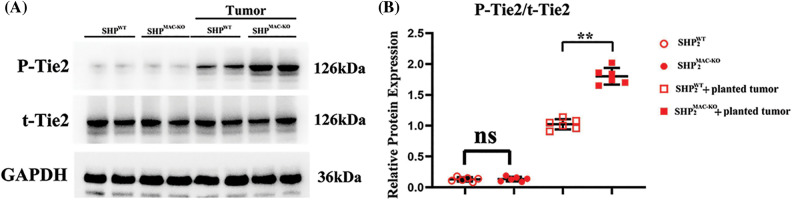
Changes in the protein expressions of Tie2 in the primary TEMs in liver CT26 CRC tissue of mice in each group. (A) The p-Tie2 and total-Tie2 protein levels in macrophages of liver tissues showed no significant differences in non-tumor environment, and macrophages’ specific deletion of SHP2 significant increased levels of phosphorylated-Tie2 but not the total-Tie2 in colorectal cancer-bearing mice; (B) Statistic data for normal western blot testing. **p < 0.01, SHP2MAC-KO mice *vs*. SHP2WT mice, N = 6/group.

**Figure S2:**
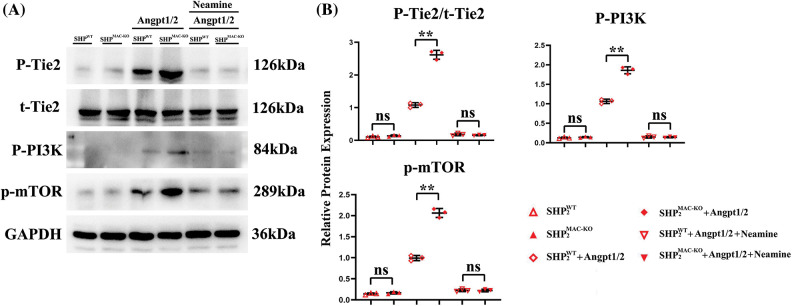
Changes in the expression of proteins related to the Tie2, PI3K, mTOR pathway in primary TEMs from the mice stimulated with angpt1/2 or neamine of different groups. (A) Primary TEMs from hepatic CT26 CRC tisssues of SHP2MAC-KO mice were treated with Angpt1/2 significantly increased the phosphorylated (p) levels of Tie2, p-PI3K, p-mTOR *vs*. SHP2WT and neamine corrected these phosphorylated trends; (B) Statistic data for western blot. **p < 0.01, primary TEMs of SHP2MAC-KO mice *vs*. primary TEMs of SHP2WT mice stimulated with Angpt1/2, N = 3/group.

The hepatic tissues of tumor-bearing mice were isolated under sterile conditions, the blood stains were washed with D-PBS, and the tissues were cut into 1mm3 tissue blocks with sterile scissors. The tissue blocks were continuously stirred and digested in a mixer containing 0.2% type IV collagenase (No. C8160, Solarbio) and 0.25% trypsin (No. T1350, Solarbio) at 37°C, using a 50 ml conical bottle and a sterile small magnetic stirring rod for 5 min each time. The supernatant was collected after the digestion was terminated with the complete medium until the tissue mass disappeared. The supernatant collected after digestion was filtered with a 200-micron mesh screen, resuscitated with a cell staining buffer (1 × PBS containing 1% BSA),and refrigerated at 4°C. Cell staining and flow sorting: adjust the cell density to 1 × 106/100 μl and add anti-F4/80-FITC (No. 11-4801-82, Invitrogen) to avoid light staining for 30 min. Cells were analyzed and sorted by BD FACSAria III, and the photomultiplier tube (PMT) and adjustment compensation (FITC *vs*. APC) were optimized by monochromatic anti-F4/80-FITC and negative control. We then remove cell fragments and clumps based on cell size. Tumor-associated macrophages (TAMs) were purified by using the regional labeling of the F4/80+ cell population. The first antibodies: SHP-2, Tie2 and p-Tie2, p-PI3K, and p-mTOR were purchased from Abcam, and these results were represented in Suppl. Figs. S1 and S2.

### Collection and storage of CRC specimens from humans

Normal colon tissues and CRC specimens from humans, n = 7/group, were cut off surgically and put in formalin at room temperature. These tissues were obtained in paraffin-embedded and sectioned into 5 μm for further immunofluorescence staining. All operation processes are under the ethical committee of Tianjin Medical University Nankai Hospital.

### Statistical analysis

SPSS 17.0 (Armonk, NY, USA) was used to build a database and carry out statistical analysis. Measurement data were represented by $\bar{\chi }$ ± s. In addition, one-way analysis of variance and *t*-test were applied to the comparison among multiple groups and between two groups, respectively. Statistically significant differences were defined by *p* < 0.05.

## Results

### Changes in the number of metastatic cancer nodules on the liver surface

**Figure 1:**
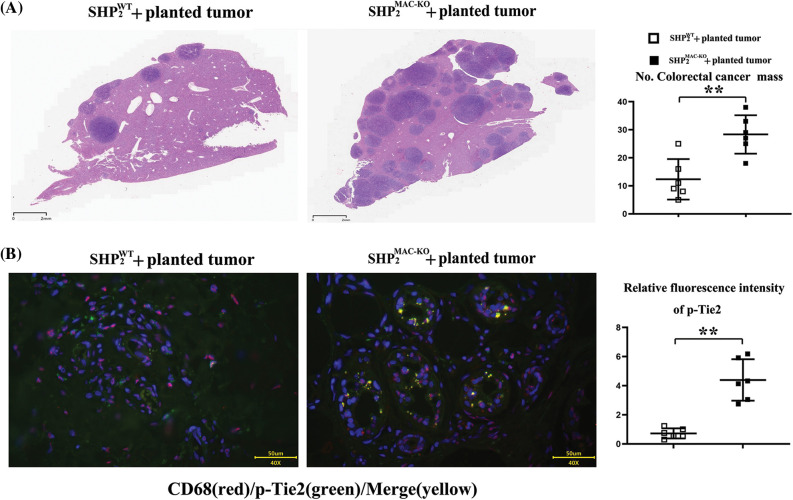
Changes of the numbers of metastatic cancer nodules in liver and the intensity of Tie2 expression. (A) Macrophages’ specific deletion of SHP2 accelerated amounts of colorectal cancer nodules in liver and the statistical data, scar bar: 2 mm; (B) Macrophages’ specific deletion of SHP2 increased the fluorescent intensity of Tie2 in CD68/macrophages marker cells and the statistical data, scar bar: 50 μm. **p < 0.01, SHP2MAC-KO mice *vs*. SHP2WT mice, N = 6/group.

Macrophages-specific SHP-2-deficient mice planted with CT26 cells displayed significant numbers of metastatic cancer nodules in the liver *vs*. wild-type mice (Fig. 1A). Our results indicated the macrophages’ SHP-2 deficiency promoted tumor metastasis in the liver.

### Changes in the fluorescence intensity of p-Tie2

**Figure 2:**
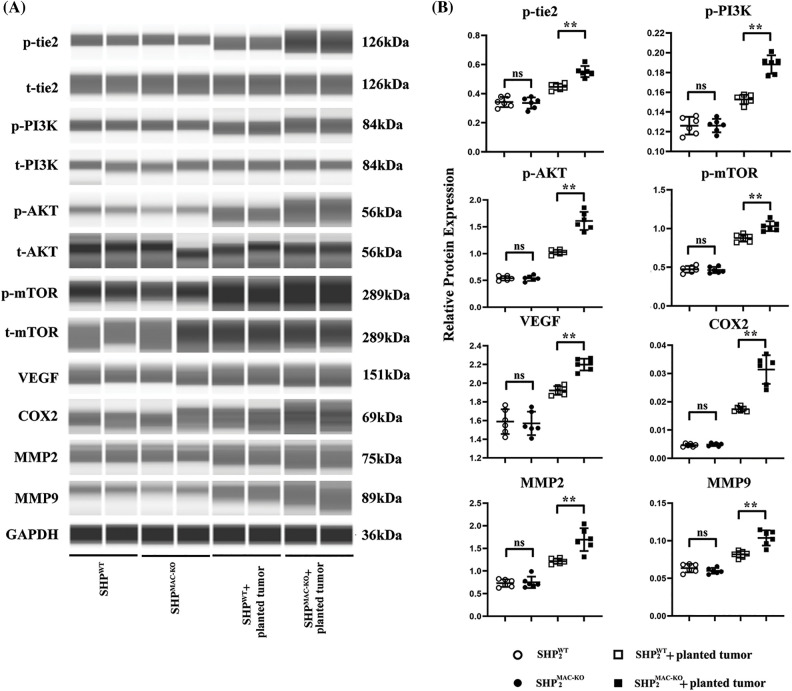
Changes in the expression of proteins related to the Ang/Tie2-PI3K/Akt/mTOR pathway in macrophages of liver tissue of mice of different groups. (A) The Tie2-PI3K/Akt/mTOR in macrophages of liver tissues showed no significant differences in non-tumor environment, and macrophages’ specific deletion of SHP2 significantly increased levels of phosphorylated Tie2-PI3K/Akt/mTOR as well as the expression of COX-2, VEGF, MMP-2/-9 in colorectal cancer-bearing mice; (B) Statistical data for Western blot. **p < 0.01, primary TEMs from tumor tissues of SHP2MAC-KO mice *vs*. SHP2WT mice, N = 6/group.

Compared with the SHP-2WT + planted tumor group, the SHP-2MAC-KO + planted tumor group obtained significantly enhanced fluorescence intensity of p-Tie2 in liver tissue (Fig. 1B), indicating the high-level expression of p-Tie2 in the liver tissue of SHP-2MAC-KO + planted tumor mice. Moreover, the CD68 (macrophages’ specific marker) positive cells were consistent with p-Tie2-positive expressing areas, indicating the SHP-2 deficiency in Tie2+ macrophages (TEMs) resulted in the excessively activated Tie-2 signals *vs*. SHP-2WT mice, data were shown in Figs. 2 and Suppl. S1 tested by digital Western blot and normal Western blot, respectively.

### SHP-2 suppression inhibits the activation of the Ang/Tie2-PI3K/Akt/mTOR pathway in the liver with CRC liver metastasis

**Figure 3:**
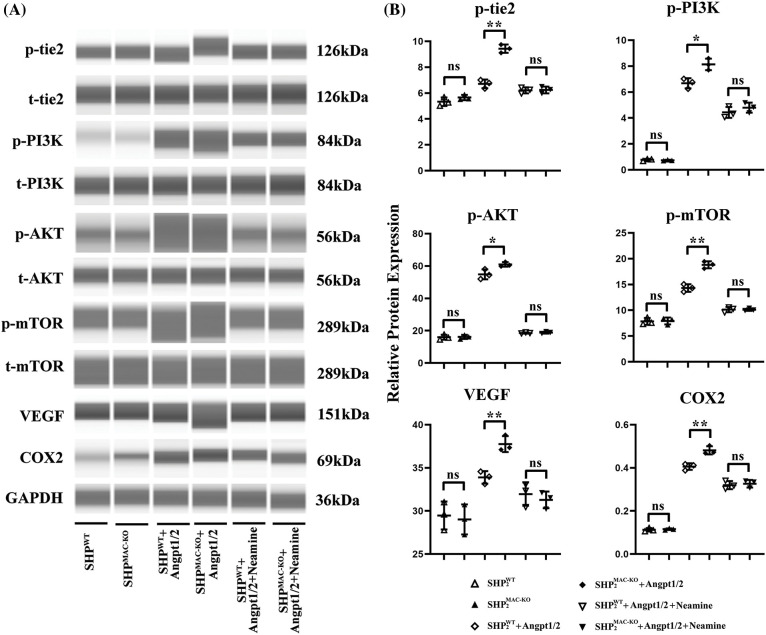
Changes in the expression of proteins related to the Ang/Tie2-PI3K/Akt/mTOR pathway in primary TEMs from the mice of different groups. (A) Primary bone marrow-derived macrophages from SHP2MAC-KO mice were treated with Angpt1/2 significantly increased the phosphorylated levels of Tie2-PI3K/Akt/mTOR *vs*. SHP2WT and neamine corrected these phosphorylated trends; (B) Statistic data for western blot. *p < 0.05, **p < 0.01, primary TEMs of tumor tissues from SHP2MAC-KO mice *vs*. SHP2WT mice stimulated with Angpt1/2, and Neamine, N = 3/group.

**Figure S3:**
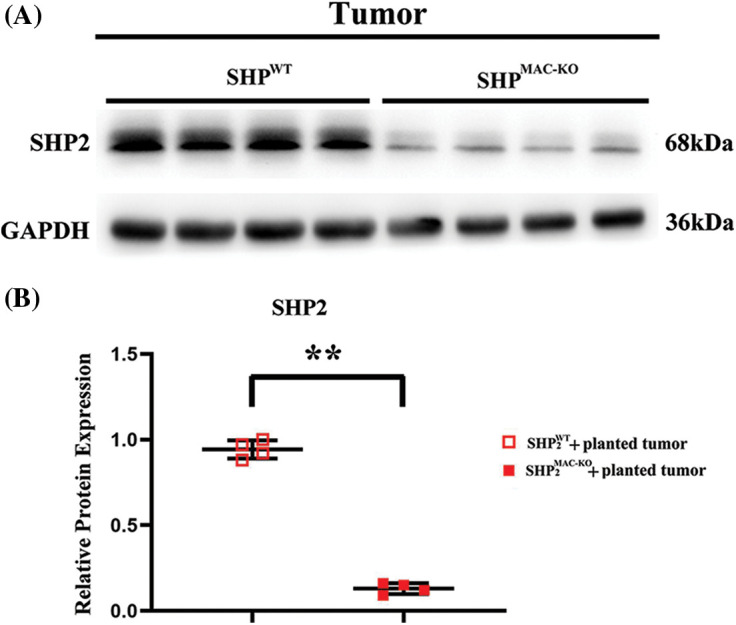
Changes in the protein expressions of SHP2 in the primary TEMs in liver CT26 CRC tissue of mice. (A) The SHP2 protein levels in TEMs showed significant differences in macrophages’ specific deletion of SHP2 and control group in colorectal cancer-bearing mice; (B) Statistic data for normal western blot testing. **p < 0.01, SHP2MAC-KO mice *vs*. SHP2WT mice, N = 4/group.

The proteins of F4/80+, Tie2+ TEMs sorting by flow cytometry were tested by digital western blot and the expression of proteins related to the Ang/Tie2-PI3K/Akt/mTOR pathway in the liver tissue of mice. As revealed by Fig. 2, after CRC liver metastasis, the expression of proteins related to the Ang/Tie2-PI3K/Akt/mTOR pathway in the Tie2+ macrophages sorted from liver tissue of SHP-2WT and SHP-2MAC-KO mice experienced a significant rise. Furthermore, compared with the SHP-2WT + planted tumor group, the SHP-2MAC-KO + planted tumor group exhibited a significant increase in p-Tie2, p-PI3K, p-Akt, p-mTOR, VEGF, COX-2, MMP2, and MMP9 in the liver tissue. These studies directly tested the differences of functions or activation of transduction signals of primary TEMs from hepatic metastatic colorectal cancer (CT26 cells) in SHP-2WT + planted tumor group, the SHP-2MAC-KO + planted tumor group as the experiments *in vivo* (Figs. 3 and Suppl. S2). The expression of SHP2 was tested by normal Western blot to identify the phenotype of SHP2MAC-KO mice and SHP2WT mice and data were shown in Suppl. Fig. S3.

### Conditional knockout of SHP-2 promoted the activation of the Ang/Tie2-PI3K/Akt/mTOR pathway in TEMs

In Fig. 3, primary Tie2+, F4/80+ TEMs were cultured *in vitro*, after Angpt1/2 was added to the TEMs of SHP-2WT and SHP-2MAC-KO mice, significantly high-level expressions were observed in p-Tie2, p-PI3K, p-Akt, p-mTOR, p-Tie2, p-PI3K, p-Akt, p-mTOR, VEGF, and COX-2. Furthermore, the expression of each protein in the SHP-2MAC-KO + Angpt1/2 group rose compared with that in the SHP-2WT + Angpt1/2 group. Under the simultaneous stimulation of Angpt1/2 + Neamine, no obvious change appeared in the expression of each protein. The primary Tie2+, F4/80+ TEMs were stimulated with Angpt1/2 and enamine for experiments *in vitro*.

### SHP-2 deficiency promoted the migration and invasion of TEMs in vitro

**Figure 4:**
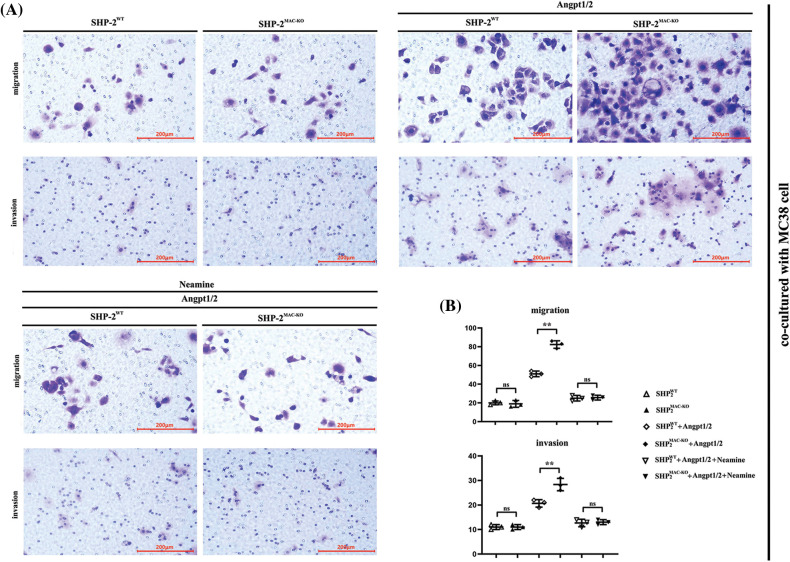

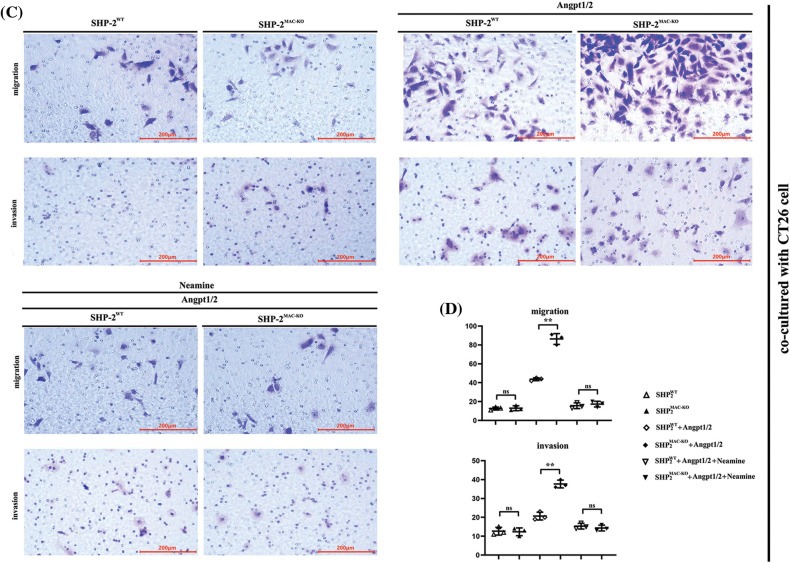
Changes in migration and invasion abilities of MC38 and CT26 cells co-cultured with primary TEMs respectively. (A) The numbers of migrated and invaded MC38 cells when co-cultured with primary TEMs of SHP2WT mice and SHP2MAC-KO mice stimulated with Angpt1/2 or neamine; (B) The statistical data of the numbers of MC38 cells; (C) The numbers of migrated and invaded CT26 cells when co-cultured with primary TEMs of SHP2WT mice and SHP2MAC-KO mice treated with Angpt1/2 or neamine; (D) Statistic data of the numbers of CT26 cells; **p < 0.01, MC38 or CT26 cells co-cultured with primary TEMs of SHP2MAC-KO mice *vs*. primary TEMs of SHP2WT mice stimulated with Angpt1/2, N = 3/group.

**Figure 5:**
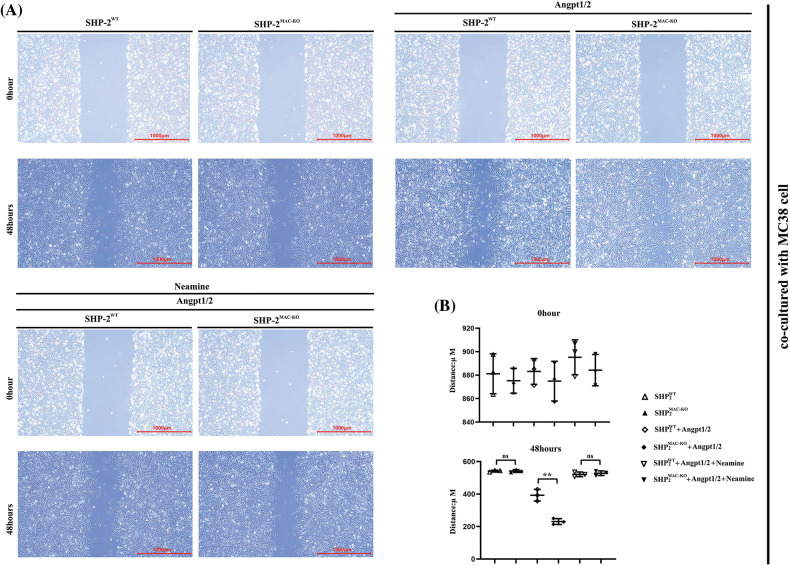

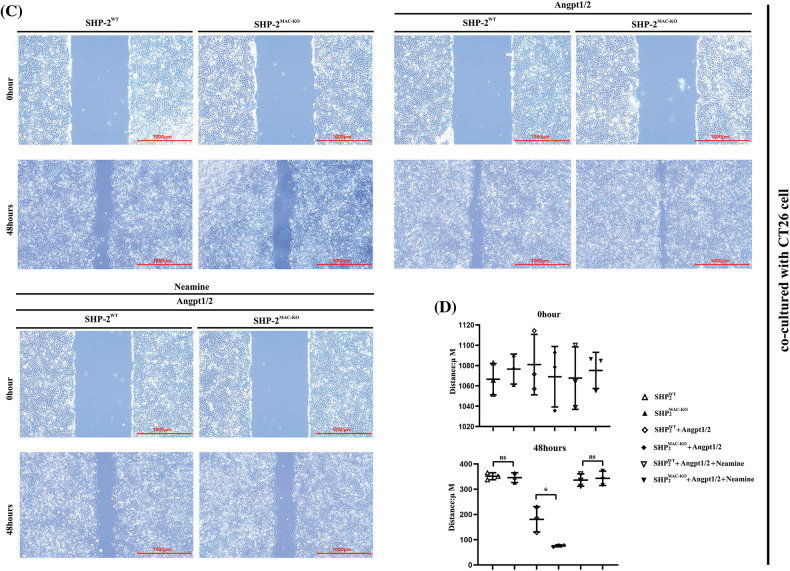
Changes in migration abilities of MC38 and CT26 cells co-cultured with TEMs tested by wound healing assay. (A) The distances of MC38 cells co-cultured with TEMs from of SHP2WT and SHP2MAC-KO mice treated with Angpt1/2 and or neamine; (B) The statistical data for MC38 cells’ migrated distances in wound healing assay; (C) The distance of migrated CT26 cells co-cultured with TEMs from of SHP2WT and SHP2MAC-KO mice treated with Angpt1/2 and or neamine; (D) The statistical data for CT26 cells’ migrated distances in wound healing assay; *p < 0.05, **p < 0.01, the migrated distances of MC38 or CT26 cells co-cultured with primary TEMs of SHP2MAC-KO mice *vs*. primary TEMs of SHP2WT mice stimulated with Angpt1/2, N = 2/group.

Transwell assay was used to explore the influence of conditional knockout of SHP-2 on the migration and invasive abilities of TEMs, co-cultured with MC38 and CT26 cells, respectively. As shown in Fig. 4, the number of cells passing through the lower chamber and the basement membrane underwent significant growth when the TEMs co-cultured with MC38 and CT26 cells, respectively, were stimulated by Angpt1/2. Moreover, the SHP-2MAC-KO + Angpt1/2 group had evident more cells passing through the lower chamber and the basement membrane than the SHP-2WT + Angpt1/2 group. However, the number of cells in this regard was subjected to no obvious change under the simultaneous stimulation of Angpt1/2 + Neamine. Moreover, the wound healing assays were performed to assess cancer cells’ migratory ability in the horizontal direction, MC38 and CT26 cells co-cultured with SHP-2MAC-KO cells showed the promoted migration ability *vs*. co-cultured with SHP-2WT cells, and enamine corrected this phenomenon (Fig. 5).

### Specific deficiency of SHP-2 in TEMs promoted tube formation of C166 cells

**Figure 6:**
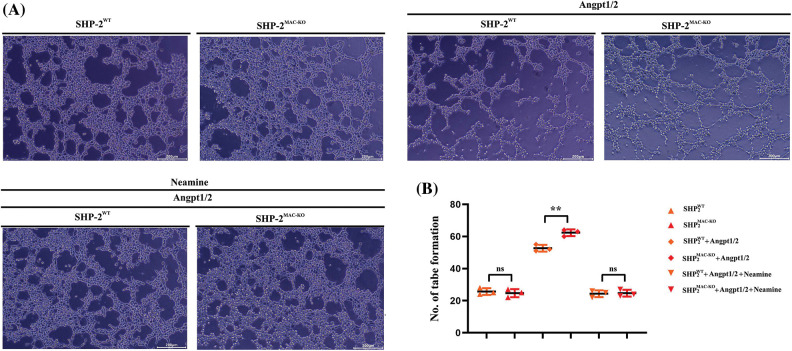
Changes in the number of blood vessels formed after the C166 cells co-cultured with TEMs. (A) The numbers of vessel/tube formations of C166 cells co-cultured with TEMs from SHP2WT and SHP2MAC-KO mice treated with Angpt1/2 and or neamine; (B) Statistic data for tube formation. **p < 0.01, the numbers of tube formations of C166 cells co-cultured with primary TEMs of SHP2MAC-KO mice *vs*. primary TEMs of SHP2WT mice stimulated with Angpt1/2, N = 3/group.

According to the results of the tube formation assay (Fig. 6), the number of newly formed blood vessels experienced obvious growth after the TEMs co-cultured with C166 cells was stimulated by Angpt1/2. Moreover, the number of newly formed blood vessels in the SHP-2MAC-KO+ Angpt1/2 group was significantly larger than that in the SHP-2WT + Angpt1/2 group. No obvious change was found in this regard under the simultaneous stimulation of Angpt1/2 + Neamine.

### SHP-2 expression was elevated in CRC than normal Human colon tissue

**Figure 7:**
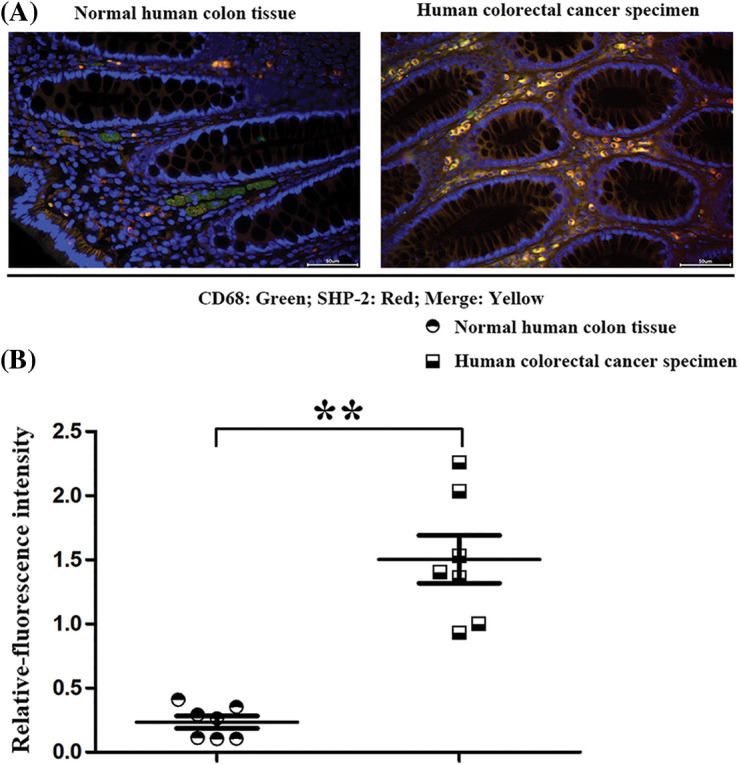
The SHP-2 expression levels in macrophages in human specimens. (A) The macrophages’ marker: CD68 (red) and SHP-2 (green) were stained by immunofluorescence double staining in normal human colon tissues and colorectal cancer tissue; (B) Statistic data for immunofluorescence intensity, **p < 0.01, N = 7/group.

The specimens of humans were collected and used for pathological evaluation of SHP-2 expression in tumor macrophages. CD68, the macrophage’ marker, and SHP-2 were used for immunofluorescence double staining to identify the co-expression of SHP-2 in macrophages. Results showed that significantly increased SHP-2 was found in macrophages in CRC than in norm colon tissue (Fig. 7). These results *in vivo* coincide with Wenbin Chen’s results *in vivo* [36].

## Discussion

CRC liver metastasis is one of the difficulties in CRC treatment. About 15%–25% of CRC patients had liver metastasis, i.e., synchronous liver metastasis. Most liver metastases cannot be radically resected in the initial stage (R0), resulting in a short survival time [37,38]. The exploration of CRC liver metastasis from the perspective of molecular biology for targeted inhibition of liver metastasis has become a current research focus of clinicians [39,40]. It was found in the present experiments that conditional knockout of the SHP-2 gene and removal of the dephosphorylation activity of SHP-2 could contribute to the activation of the Ang/Tie2-PI3K/Akt/mTOR pathway in TEMs, thus strengthening the tumor micro angiogenesis in the TME and facilitating CRC liver metastasis.

**Figure 8:**
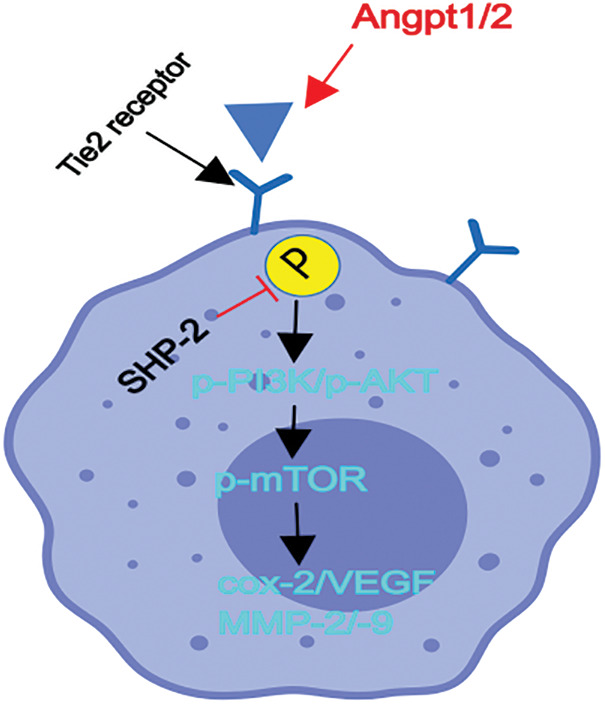
Structure diagram. Conditional knockout of SHP-2 in TEMs and the deletion of the SHP-2 promoted the activation or phosphorylation of the Ang/Tie2-PI3K/Akt/mTOR pathway in TEMs, thereby enhancing down-stream proangiogenic factor: COX-2, VEGF, MMP-2/-9 and resulting into the tumor microangiogenesis and facilitating CRC’ liver metastasis.

The imbalance between tyrosine phosphatase (mainly SHP1, SHP2) and tyrosine kinase (such as SYK, Src, Lyn, etc.) play critical roles in various diseases. SHP1 could effectively and directly dephosphorylate the tyrosine kinases, its roles were very simple and unitary, nevertheless, SHP2 could dephosphorylate or even phosphorylated tyrosine kinases dependent on the cellular types or diverse disease conditions, SHP2 plays the effects of the complex, volatile or equivocal roles in the progression of diseases [18–22]. Tie-2 is a receptor tyrosine kinase, the activated or phosphorylated form of Tie-2 is controlled by tyrosine phosphatase SHP2, however, the relationship and detail mechanisms of SHP-2 in TEMs remain unclear, thereby the conditional-knockout SHP-2 in myeloid or monocytes/macrophages were used in CT26 tumor-planted mouse model and primary F4/80, Tie2 + TEMS were sorted by flow cytometry. These primary TEMs from hepatic CT26 tumors were used for experiments *in vivo* and *in vitro*. Firstly, through the construction of SHP-2-deficient mouse models of CRC liver metastases, it was found that specific SHP-2-deficient mice had significantly more metastatic cancer nodules on the liver surface than wild-type mice, and a high-level expression of p-Tie2 was observed in the liver tissue of SHP-2MAC-KO + planted tumor mice. These findings are in line with the previous literature that the deletion of SHP-2 can activate the phosphorylation of Tie2 [34]. Furthermore, the increase in the number of metastatic cancer nodules on the liver surface of mice due to the deletion of SHP-2 is a new finding in this research. For this reason, the specific mechanisms of SHP-2 influencing the CRC liver metastasis of mice were further explored. As the main channel for TEMs to promote tumor micro-angiogenesis and tumor immunosuppressive microenvironment, the PI3K/Akt/mTOR pathway plays a role in controlling such biological processes as cell proliferation, differentiation, migration, apoptosis, and adhesion [41–43]. The results of the present study showed that after CRC liver metastasis, the SHP-2MAC-KO + planted tumor group obtained significantly higher levels of p-Tie2, p-PI3K, p-Akt, p-mTOR, VEGF, COX-2, MMP2, MMP9 in the liver tissue than the SHP-2WT + planted tumor group. It indicated that the deletion of SHP-2 promotes the activation of the Ang/Tie2-PI3K/Akt/mTOR pathway in the liver tissue of mice with CRC liver metastasis, indicating the SHP-2 dephosphorylated Tie2, a receptor tyrosine kinases (RTK), which is activated by angpt1/2 directly and results in the phosphorylated form of Tie-2 [44,45]. As reported in recent studies, while promoting tumor immune escape, the tumor immunosuppressive microenvironment also plays a crucial role in benefiting tumor micro-angiogenesis. Immunosuppressive inflammatory cells secrete MMPs to promote the matrix remodeling outside tumors [42], secrete pro-angiogenic factors including VEGF, transforming growth factor-β (TGF-β), and EGF, break the balance and homeostasis of blood vessels in the tumor tissue and enhance the sprouting and junctions of peripheral vessels, thus creating favorable conditions for the tumor micro-angiogenesis [7,43]. In the present experiments, the increased expression of VEGF, COX-2, MMP2, and MMP9 protein in the liver tissue of mice with CRC liver metastasis after SHP-2 knockout revealed that the deletion of SHP-2 may promote the tumor micro-angiogenesis. The structure diagram is shown in Fig. 8.

Hence, *in vitro*, firstly the primary TEMs, from hepatic CT26 tumor tissues were cultured and stimulated with Angpt1/2 to activate the Tie receptor in TEMs, which were carried out to examine the mediating role of the down-stream signals of Tie2: PI3K/Akt/mTOR pathway in the influence of macrophages’ SHP-2-cKO on CRC liver metastasis of mice and the influence of SHP-2 on tumor microvascular remodeling. The results showed that the expression of proteins related to the Ang/Tie2-PI3K/Akt/mTOR pathway, the number of cells passing through the lower chamber and the basement membrane, and the number of blood vessels formed by cells in the SHP-2MAC-KO + Angpt1/2 group were all increased as compared to those in the SHP-2WT + Angpt1/2 group. Moreover, Neamine tetrahydrochloride is an anti-angiogenesis agent targeting angiogenin, which inhibited the roles of Angpt1/2, and enamine-stimulation was used as a corrective experiment *in vitro* to rectify the effects of Angpt1/2. It suggested that the knockout of SHP-2 enhances the migration and invasion abilities of TEMs and promotes tumor micro-angiogenesis. In contrast, under the simultaneous stimulation of Angpt1/2 + Neamine, no significant change was found in these indexes, further indicating that the influence of SHP-2 on the migration and invasion abilities of TEMs and tumor micro-angiogenesis is mediated by the PI3K/Akt/mTOR signaling pathway. To sum up, conditional knockout of SHP-2 promotes the activation of the Ang/Tie2-PI3K/Akt/mTOR pathway in TEMs, thereby strengthening tumor micro-angiogenesis in the TME and facilitating CRC liver metastasis. Eventually, we tested the expression of SHP-2 in tumor-associated macrophages and the results showed that SHP-2 expression was significantly increased in human CRC tissues *vs*. human normal colonic tissue, which is consistent with previous studies of Wenbin Chen’s previous studies [36]. In conclusion, SHP-2 in TEMs suppressed the activation or phosphorylated-Tie2 and consequently deactivated down-stream of PI3K-Akt-mTOR and resulted in the inhibited expression of angiogenic factor: VEGF, COX-2, MMPs, and eventually protect the tumor angiogenesis in CRC.

## Data Availability

Raw data are available from corresponding author on reasonable request.
